# SMARCA4-deficient undifferentiated tumor with high quality of life and far exceeding predicted survival: A case report

**DOI:** 10.1097/MD.0000000000039045

**Published:** 2024-08-02

**Authors:** Juan Lin, Qi Ren, Binbin Liu

**Affiliations:** a Intensive Care Unit, Zhejiang Hospital, Hangzhou, Zhejiang, China; b Gastroenterology Department, Zhejiang Hospital, Hangzhou, Zhejiang, China.

**Keywords:** immunohistochemistry, predicting survival, quality of life, *SMARCA4*, SMARCA4-deficient undifferentiated tumor

## Abstract

**Rationale:**

SMARCA4-deficient undifferentiated tumor (SMARCA4-UT) is a recently reported rare malignancy that can rapidly metastasize to tissues and organs throughout the body. The tumor is characterized by a lower response to platinum-based chemotherapy. More regrettably, the mean survival time of patients with this disease after diagnosis is only 4 to 7 months.

**Patient concerns:**

A 58-year-old man was admitted to a hospital for fatigue, sudden syncope, and a mass-like shadow of his left upper lobe demonstrated by a pulmonary computed tomographic. Based on his subsequent clinical and pathological features, he was highly suspected of SMARCA4-UT.

**Diagnoses:**

Combined with next-generation sequencing genetic testing and immunohistochemical examination results, the patient was diagnosed with SMARCA4-UT.

**Interventions:**

The patient received a left upper lobectomy and lymph node dissection, four-course chemotherapy divided into 8 sessions with the use of paclitaxel simply, and a proper post-discharge self-care.

**Outcomes:**

The patient’s operation and chemotherapy were all successful and he maintained a high quality of life after surgery that far exceeded his predicted survival.

**Lessons:**

Early diagnosis, higher education level, attention to the disease and complications, reducing chemotherapy damage, adequate nutrient intake, relieving symptoms, controlling depression, and maintaining immunity and the ability to perform activities of daily living may all be the positive factors that can prolong the survival of patients with SMARCA4-UT.

## 1. Introduction

*SMARCA4* is a nucleoprotein-related gene on chromosome 19p13 that encodes an ATP-dependent catalytic subunit of SWI/SNF chromatin-remodeling complexes that participate in regulating chromatin structure and gene expression by supplying energy.^[1]^ The high-frequency *SMARCA4* mutation is involved in the pathogenesis of 2 subtypes of tumors: SMARCA4-deficient non-small cell lung cancer and SMARCA4-undifferentiated tumors (SMARCA4-UT) that harbor an undifferentiated phenotype close to those of malignant rhabdoid tumors.^[2]^ Fewer than 100 SMARCA4-UT cases have been reported to date, and it is a rare malignancy characterized by rapid progression and poor prognosis.^[3,4]^ SMARCA4-UT presents as large, compressive, and infiltrative mediastinal, lung, and/or pleural masses in middle-aged male smokers.^[5]^ Most patients generally experience a series of compression- or infiltration-related symptoms such as dyspnea, pain, cough, hemoptysis, and superior vena cava syndrome.^[2]^ SMARCA4-UT diagnosis depends on its distinct clinical and pathological features. Furthermore, *SMARCA4* deficiency is easily determined by immunohistochemistry (IHC), which demonstrates the loss of nuclear expression of *SMARCA4* protein in the tumor cell nuclei.^[6]^ SMARCA4-UT demonstrates a relevant metastatic tendency and a lower response to platinum-based chemotherapy,^[7–9]^ and the mean patient survival time is only 4 to 7 months after definite diagnosis.^[5]^ Here, we discuss a male patient with SMARCA4-UT who maintained a high quality of life after surgery that far exceeded his predicted survival.

## 2. Case presentation

A 58-year-old man, with no medical, family, and psychosocial history, was admitted to the thoracic surgery department of our hospital on May 22, 2021, for fatigue, sudden syncope, and a mass-like shadow of his left upper lobe demonstrated by a chest computed tomographic (CT) image (May 19, 2021, Fig. 1). No family history of cancer was reported; otherwise, the patient reported a current tobacco smoking habit (approximately 30 pack-years) and alcohol consumption (approximately 150 mL/day). On May 24, 2021, a left upper lobectomy and lymph node dissection were planned for the patient after a preoperative discussion.

**Figure 1.**
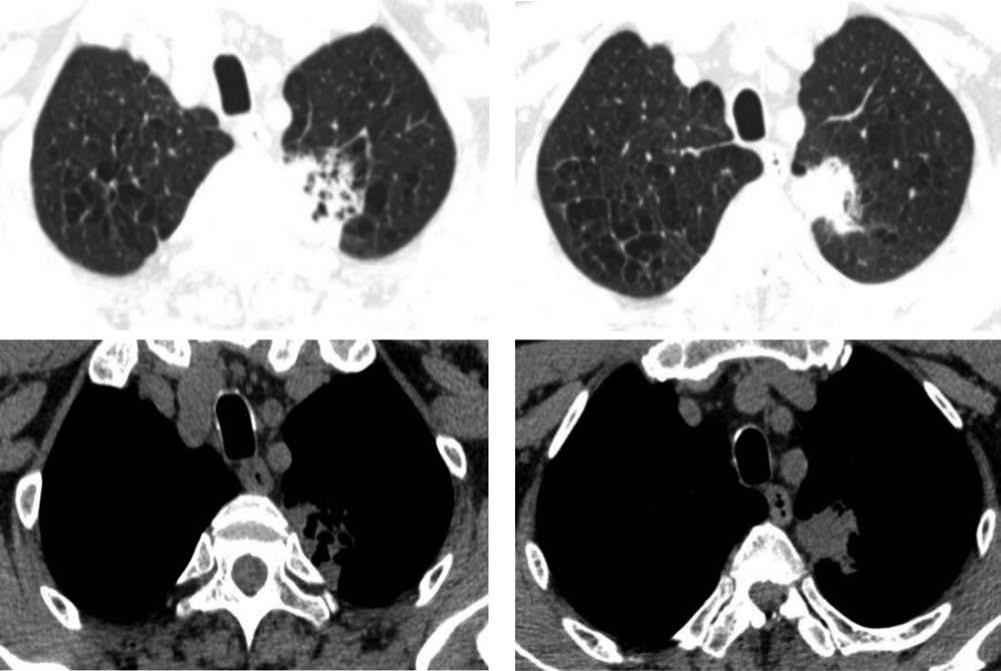
The result of a chest CT of the patient in our hospital on May 19, 2021.

The operation was performed on May 25, 2021, during which the tumor (approximately 3.5 cm × 3.0 cm × 3.0 cm) was found in the left tip of the lung. The cytopathological result demonstrated showed suppuration with extensive necrosis. The self-examination result of the lung cut margin was negative. The 2 enlarged lymph nodes in group 4L, one of which contained some heterogeneous cells, were examined. A radical lobectomy and lymph node dissection of groups 7, 9, 10, and 12 in his chest were performed. The intraoperative procedure went well, and his bleeding volume was approximately 120 mL. Cefuroxime sodium was used after the operation and was discontinued on May 26, 2021.

**Figure 2.**
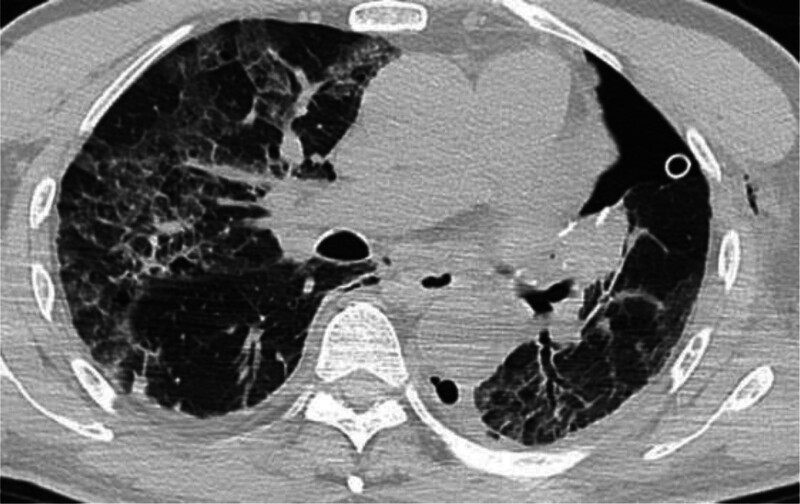
The results of a chest CT in our hospital on May 27, 2021.

On the morning of May 27, 2021, the patient had a difficult expectoration, and his transcutaneous oxygen saturation gradually dropped to 88% to 90%. A blood gas analysis demonstrated an arterial partial oxygen pressure of 58.6 mm Hg. A blood routine examination revealed a white blood cell count of 21 × 10^9^/L, a neutrophilic granulocyte percentage of 86.9%, a hypersensitive C-reactive protein of 116.30 mg/L, and a procalcitonin of 0.14 ng/mL. A chest CT revealed a small fluid pneumothorax on the left side, with a small accumulation of gas under the left chest wall. Furthermore, the 2 lungs contained scattered exudation. A small amount of fluid was accumulated on both sides of the chest (Fig. 2), and a medium amount of light bloody fluid was extracted from the left thoracic drainage tube.

On May 28, 2021, the patient’s transcutaneous oxygen saturation dropped further to only 80% to 85%. After an endotracheal intubation, he was transferred to the intensive care unit to receive mechanical ventilation, an anti-inflammatory, and anti-infection treatment. On May 30, 2021, the patient was weaned off the ventilator and extubated on June 1, 2021. On June 3, 2021, he was transferred back to the thoracic surgery department. There, the patient continued to receive a closed thoracic drainage and anti-infection treatment. His doctors adjusted the antibiotic level according to his status and inflammatory index.

The patient’s first IHC result (June 3, 2021) was as follows: TTF-1 (–), Napsin A (–), CK7 (–), CK5/6 (–), P40 (–), ALK (–), P53 (partly +), E-cadherin (+), Ki67 (+, 35%), CK (Pan) (+), vimentin (+), CD56 (–), CD34 (+), and D2-40 (–). Subsequently, the IHC result was SALL4 (+). The pathological result indicated (June 3, 2021, Fig. 3) was as follows: (1) the wedge resection specimen of his left upper lung was a poorly differentiated malignant tumor. The tumor cells were diffuse and flaky-like with obvious necrosis, oval epithelioid, abundant cytoplasm, vacuolar nucleoli, and more lymphoplasmic cells. Therefore, a sarcomatoid carcinoma or undifferentiated tumor was considered in combination with the manifestation of his histomorphology and IHC results.^[2,10,11]^ (2) There was no metastasis in the group 7, 9, 10, and 12 lymph nodes. However, there was a metastasis in 1 (of 2) parabronchial margin lymph nodes.

**Figure 3.**
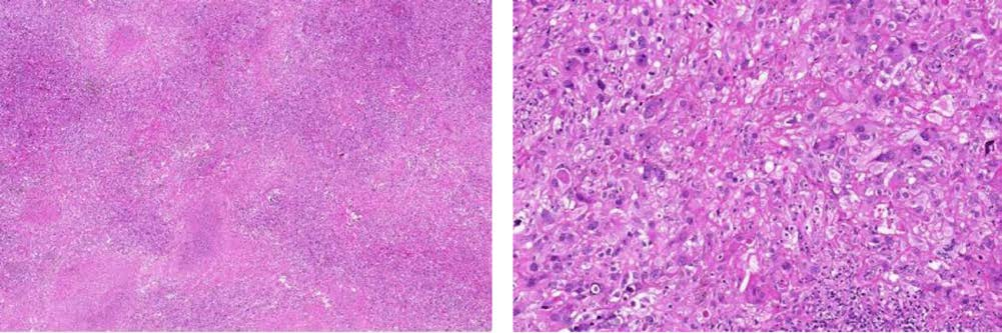
The pathological result of this patient.

On June 9, 2021, consultation with the oncologists yielded the definitive diagnosis of poorly differentiated sarcomatoid carcinoma with bronchial lymph node metastasis. Combined with next-generation sequencing genetic testing and immunohistochemical examination results, the patient’s diagnosis was consistent with SMARCA4-UT. The driver gene mutation testing result was negative (Table 1); accordingly, the patient was advised to receive adjuvant chemotherapy combined with antiangiogenic therapy.^[12]^ However, he refused the chemotherapy at our hospital and was discharged on June 22, 2021.

**Table 1:** Detection of point mutations, insertion or deletion of small fragments.

Gene	Transcript	Base change	Amino acid change	Functional region	Mutation frequency
FAT2	NM_001447.2	c.1984C > A	p.P662T	EX1	39.6%
FGFR4	NM_213647.1	c.1285A > T	p.S429C	EX10	38.6%
SMARCA4	NM_003072.3	c.2321A > T	p.N774I	EX16	32.3%
HGF	NM_000601.4	c.2119G > C	p.V707L	EX18	31.7%
WWP1	NM_007013.3	c.664G > C	p.A222P	EX8	31.0%
NOTCH1	NM_017617.3	c.4507G > T	p.G1503C	EX25	30.9%
SETD2	NM_014159.6	c.7407A > T	p.K2469N	EX19	29.0%
TSC2	NM_000548.3	c.1739C > T	p.A580V	EX17	26.2%
INHBA	NM_002192.2	c.686G > T	p.R229L	EX3	25.2%
MLL2	NM_003482.3	c.8552G > T	p.G2851V	EX34	24.4%
GRIN2A	NM_001134407.1	c.1006C > A	p.P336T	EX3	24.3%
CDK8	NM_001260.1	c.819G > T	p.M273I	EX8	23.5%
BBS9	NM_198428.2	c.566G > C	p.R189P	EX6	19.1%
LRP1B	NM_018557.2	c.2668C > A	p.Q890K	EX17	15.4%
LRP1B	NM_018557.2	c.7985C > A	p.S2662Y	EX49	14.6%
LRP1B	NM_018557.2	c.9815–1G > T	—	IVS61	13.4%
FAP	NM_004460.2	c.620C > A	p.A207D	EX9	13.1%
NOTCH4	NM_004557.3	c.3326G > C	p.G1109A	EX21	12.4%
CYLD	NM_001042355.1	c.1070G > A	p.G357E	EX7	10.8%

Approximately 2 months after being discharged, the patient took the advice of the doctor at another oncology hospital and received four-course chemotherapy divided into 8 sessions with the use of paclitaxel simply (170 mg, IVGTT, q3w; pre-chemotherapy antiemetic: palonosetron, without antiangiogenic therapy). The most common adverse effects in patients who use paclitaxel include reduced red blood cell, platelet, and white blood cell counts, numbness, tingling, pain, or weakness in the hands and feet, fatigue, hair loss, and nausea.^[13]^ Currently, our patient has a positive mental state and is emotionally stable without obvious discomfort. His appetite, sleep, and blood test results are all normal. His weight was 72.5 kg (body mass index [BMI]: 24.79 kg/m^2^), and his quality of life returned to the favorable status before the operation. The patient’s Eastern Cooperative Oncology Group performance status score was 0 to 1,^[14]^ and he could move freely and engage in light physical activity, general housework, and office work. He was keen on growing vegetables and flowers, and his mobility was normal. His regular reviews revealed no significant tumor recurrence, and his last pulmonary CT image (May 16, 2023) only showed a partial thickening of the left hilum, which was identified as a postoperative change.

## 3. Discussion

Several studies have confirmed that frailty is an independent risk factor for the poor prognosis of patients with cancer, which often leads to a series of health problems, such as toxication and chemoradiation side effects.^[15,16]^ The incidence of frailty in patients with malignancy is 6% to 86% and can be as high as 31% in patients with lung cancer.^[17]^ A meta-analysis of the influencing factors of frailty in patients with cancer in China divided the factors into demographic (age, gender, educational level, and marital status), physical (complications, BMI, hemoglobin, albumin, nutritional status, and symptoms), and psychological or social factors (depression status and Instrumental Activity of Daily Living [IADL] score).^[18]^ Furthermore, economic status is a factor that should not be overlooked.

Advanced age is an important risk factor for frailty in patients with cancer, and the risk increases significantly with increasing age.^[19]^ A patient’s education level is another important factor in frailty and is generally directly proportional to their income and social status. Patients with high education levels can acquire relatively more medical resources and focus more on their health. Our patient is 58 years old and has a bachelor’s degree, which was a high education level for his time. He is also an armed forces veteran and retired civil servant with a relatively stable income and a certain social status. According to the patient, he paid more attention to his health management in daily life, underwent a regular physical examination every year, could correctly address detected health issues, and actively sought medical support such as dynamic physical condition assessment. Currently, the patient can conscientiously monitor blood pressure changes at home daily, regularly take medicine according to his doctor’s advice, maintain normal living habits, sleep and wake up early, have a nap of 0.5 to 1 hour daily, participate in moderate physical exercise, follow a balanced diet, and had quit smoking and drinking.

The presence of other diseases is a potential factor for the increased risk of frailty in patients with cancer. Previous studies confirmed a common pathogenesis between diseases and frailty, and active treatment and management of current diseases can delay the development of frailty.^[20]^ The patient had an increased lung infection and was admitted to the intensive care unit during hospitalization, which dimmed his prognosis. The doctors stated that the patient also had hypertension and hyperlipidemia and could take medicine regularly to control his blood pressure and blood lipid in an ideal range. To ensure that the patient had sufficient energy to tolerate the chemotherapy, he received it 2 months after the lung cancer radical surgery, when he returned to a better physical condition. Furthermore, the patient was involved in the chemotherapy formulation. The conventional plan was adjusted to divide the single-dose course in 2. Therefore, the four-course chemotherapy was divided into 8 sessions to reduce the damage to the patient’s body to maintain a better physical and mental state. The patient had no chemotherapy-related complications and felt comfortable psychologically and physically. The approach ensured a stable chemotherapy and increased his confidence in dealing with the chemotherapy and disease. The patient also actively responded to his diseases in their controllable state. At the end of 2022, the patient had a low fever for approximately 3 days (temperature: 37.5 °C) due to COVID-19. Although he had no complications, such as chest tightness and shortness, he was still actively hospitalized for treatment. In March 2023, the patient actively underwent another operation to treat kidney stones and hydronephrosis to prevent complications such as renal impairment.

Patients with high BMI (especially BMI > 30.0 kg/m^2^) developed frailty more frequently than patients with normal BMI.^[21]^ Additionally, 35.0 g/L albumin is an independent protective factor against frailty in patients with cancer, and low nutritional status is another important risk factor for frailty. Thus, increasing patients’ nutrition intake to improve their nutritional status can reduce the incidence of frailty.^[22–24]^ The patient is a retired civil servant with a stable income and good family economic condition. His wife paid attention to the nutrition collocation and changed his diet daily to ensure nutrition intake. Furthermore, the patient maintained normal stomach intake and alternately supplemented with cordyceps and sea cucumber weekly, combined with a thymus pentapeptide injection to improve immunity. The patient’s weight after chemotherapy was approximately 10 kg heavier than before the operation.

The accompanying symptoms of patients with cancer are also a cause of their frailty. The symptoms include nervous, digestive, and respiratory symptoms and can seriously affect patients’ activity and dietary intake, aggravating frailty. Thus, sufficient attention and early intervention are necessary to reduce such symptoms, alleviate frailty, and improve the quality of life, prolonging survival. In addition to the careful care and attention to the treatment of accompanying symptoms during the hospitalization, the patient also lived in the suburbs, away from noise after being discharged from the hospital. The surrounding quiet and beautiful environment provided the objective conditions for restful sleep and cultivating his morale, made him feel comfortable, and provided a favorable place for daily walking and exercise. At discharge, the patient continued to complain of fatigue and transient chest acupuncture pain. After 2 months of home care, he had no obvious accompanying symptoms. Therefore, his quality of life was improved, and his survival was prolonged.

Emotion affects the prognosis of patients with cancer, and depression is a major risk factor for frailty. A systematic review and meta-analysis from Europe indicated a reciprocal interaction between depression and frailty. The interaction indicates that an increased prevalence and incidence of the 2 conditions are mutually associated and that depression and frailty are mutual development risk factors.^[25]^ The self-life attitude is also a part of “self-healing.” The patient is a veteran and has a strong psychological strength. He developed delirium while in hospital, but it was rapidly relieved in his family members’ company. After discharge, the patient’s family was harmonious, his wife accompanied him throughout the process, and his family members focused more attention on his condition. With their utmost encouragement and support, the patient did not experience anxiety, depression, or other mental issues during his recovery period.

The IADL score reflects an important part of the ability to perform activities of daily living and affects frailty. A higher IADL score indicates that a patient is less likely to develop frailty.^[26]^ Our patient had normal daily life and social skills, such as driving, shopping, cooking, performing housework and financial management, and taking medication, and had no defects in daily life activities.

Finally, SMARCA4-UT is a rapidly metastatic aggressive tumor. Therefore, early diagnosis is critical for the prognosis of patients with SMARCA4-UT. The patient fainted twice within a short period due to fatigue and sought medical treatment immediately. The early diagnosis of the issue enabled the administration of active therapeutic intervention.

## 4. Conclusions

SMARCA4-UT is relatively rare and dangerous. Thus, a specific diagnosis should be established to fight for treatment opportunities as early as possible. Meanwhile, higher education level, attention to the disease and complications, reducing chemotherapy damage, adequate nutrient intake, relieving symptoms, controlling depression, and maintaining immunity and the ability to perform activities of daily living may all be the positive factors that protect against frailty in patients with SMARCA4-UT.

## Author contributions

**Conceptualization:** Juan Lin.

**Formal analysis:** Qi Ren.

**Supervision:** Binbin Liu.

**Writing – original draft:** Juan Lin, Qi Ren.

**Writing – review & editing:** Binbin Liu.
